# Can quantifying morphology and TMEM119 expression distinguish between microglia and infiltrating macrophages after ischemic stroke and reperfusion in male and female mice?

**DOI:** 10.1186/s12974-021-02105-2

**Published:** 2021-02-22

**Authors:** Kimberly F. Young, Rebeca Gardner, Victoria Sariana, Susan A. Whitman, Mitchell J. Bartlett, Torsten Falk, Helena W. Morrison

**Affiliations:** 1 College of Nursing, University of Arizona, 1305 N. Martin Ave., Tucson, AZ 85721 USA; 2 Current affiliation: Department of Psychology, University of Arizona, 1503 E University Blvd., Tucson, AZ USA; 3 University of Arizona Evelyn F. McKnight Brain Institute, 1333 N. Martin Ave., Tucson, AZ USA; 4 College of Science, University of Arizona, 1040 4th St., Tucson, AZ USA; 5 College of Medicine, Department of Neurology, University of Arizona, 1501 N. Campbell Ave., Tucson, AZ USA; 6 College of Medicine, Department of Pharmacology, University of Arizona, 1501 N. Campbell Ave., Tucson, AZ USA

**Keywords:** Ischemic stroke, Microglia, Sex differences, Transmembrane protein 119

## Abstract

**Background:**

Ischemic stroke is an acquired brain injury with gender-dependent outcomes. A persistent obstacle in understanding the sex-specific neuroinflammatory contributions to ischemic brain injury is distinguishing between resident microglia and infiltrating macrophages—both phagocytes—and determining cell population-specific contributions to injury evolution and recovery processes. Our purpose was to identify microglial and macrophage populations regulated by ischemic stroke using morphology analysis and the presence of microglia transmembrane protein 119 (TMEM119). Second, we examined sex and menopause differences in microglia/macrophage cell populations after an ischemic stroke.

**Methods:**

Male and female, premenopausal and postmenopausal, mice underwent either 60 min of middle cerebral artery occlusion and 24 h of reperfusion or sham surgery. The accelerated ovarian failure model was used to model postmenopause. Brain tissue was collected to quantify the infarct area and for immunohistochemistry and western blot methods. Ionized calcium-binding adapter molecule, TMEM119, and confocal microscopy were used to analyze the microglia morphology and TMEM119 area in the ipsilateral brain regions. Western blot was used to quantify protein quantity.

**Results:**

Post-stroke injury is increased in male and postmenopause female mice vs. premenopause female mice (*p* < 0.05) with differences primarily occurring in the caudal sections. After stroke, the microglia underwent a region, but not sex group, dependent transformation into less ramified cells (*p* < 0.0001). However, the number of phagocytic microglia was increased in distal ipsilateral regions of postmenopausal mice vs. the other sex groups (*p* < 0.05). The number of TMEM119-positive cells was decreased in proximity to the infarct (*p* < 0.0001) but without a sex group effect. Two key findings prevented distinguishing microglia from systemic macrophages. First, morphological data were not congruent with TMEM119 immunofluorescence data. Cells with severely decreased TMEM119 immunofluorescence were ramified, a distinguishing microglia characteristic. Second, whereas the TMEM119 immunofluorescence area decreased in proximity to the infarcted area, the TMEM119 protein quantity was unchanged in the ipsilateral hemisphere regions using western blot methods.

**Conclusions:**

Our findings suggest that TMEM119 is not a stable microglia marker in male and female mice in the context of ischemic stroke. Until TMEM119 function in the brain is elucidated, its use to distinguish between cell populations following brain injury with cell infiltration is cautioned.

**Supplementary Information:**

The online version contains supplementary material available at 10.1186/s12974-021-02105-2.

## Introduction

Ischemic stroke is a global concern as the burden of stroke continues to rise with few effective prevention strategies or treatments on the horizon [1]. In the USA, the age-standardized prevalence of ischemic stroke is among the highest in the world and a significant cause of mortality and morbidity [1, 2]. Males have a higher age-specific incidence of ischemic stroke than women; however, women experience an increase in stroke severity and incidence postmenopause [3–5]. As such, biological sex and menopause status are important variables to consider in stroke studies that investigate gender-based differences [2, 6–8]. Post-stroke neuroinflammation includes a complex, multi-faceted, and often sex-specific cellular response to injury that contributes to brain recovery and repair after ischemic stroke [9–15]. While potent neuroinflammatory responses can exacerbate injury in the acute phase [16] and, recently discovered, are slow to resolve in the brain [17, 18], neuroinflammatory and microglial responses also contribute to brain recovery by influencing brain remodeling and repair [19–21].

Microglia are the resident brain immune cells and a key player in the neuroinflammatory response following an ischemic stroke [12, 22, 23]. Infiltrating macrophages/neutrophils also contribute to inflammatory responses in the brain; however, the timing of their arrival, numbers, and distinct functions are often highly debated [22, 24–28]. Much work has previously established the role of sex and sex hormones on post-stroke brain injury outcomes (reviewed by [7, 29]), the sex-specific nature of microglia [30–32], and the sex differences in microglial responses to ischemic stroke [12, 33, 34]. However, it remains that due to the common lineage between microglia and macrophages [35, 36], it is difficult to distinguish between these populations, further complicating our understanding of cell-specific neuroinflammatory responses. This limitation impairs our ability to provide therapies that precisely target key contributors to injury resolution vs. exacerbation in male and female stroke patients.

Morphology, parenchymal distribution, and a unique transcriptional profile are key distinguishing characteristics between these two similar cell populations [36–39]. Microglia are highly ramified cells that become less ramified in proximity to an injury [37], whereas historically, macrophages have been consistently described as round/amoeboid. Challenging this notion, macrophages gradually transformed into ramified cells after reprograming in a model of neonatal stroke [40]. While not uniquely located to the central nervous system [41–44], transmembrane protein 119 (TMEM119) is a protein that, in the brain, is specific to microglia and identified as a useful tool for segregating microglia from macrophages [45]. However, its function and involvement in the brain’s injury response are yet unknown [46–48]. Its usefulness as a unique microglia protein marker was investigated and shown to have stable and specific protein expression in microglia in response to lipopolysaccharide (LPS)-induced neuroinflammation and optic nerve crush [45]. However, recent data may contradict the stability of TMEM119 in the context of injury by illustrating that *Tmem119* transcripts are decreased post-injury in a model of intracerebral hemorrhage [49]. The use of TMEM119 to distinguish between microglia and macrophage cell populations in preclinical models of adult ischemic stroke is limited [50]. Combining two in situ methodologies, morphological analysis [51] and TMEM119 immunofluorescence and protein expression [45], our purpose was to definitively identify microglial and macrophage populations regulated by ischemic stroke. Our secondary purpose was to test for the differences in sex and, in females, the effect of menopause on microglia/macrophage cell populations after an ischemic stroke. Utilizing a 60-min ischemia and 24-h reperfusion stroke protocol, we investigated if microglia transitioned into less ramified cells in proximity to the infarct similarly among our sex groups—male, premenopause, and postmenopause mice. The TMEM119 immunofluorescence was also investigated among the sex groups, to test how far the different cell populations might extend past the infarct border.

## Methods

### Animals

All animal handling and experiments were performed according to the methods approved by and in compliance with the University of Arizona Institutional Animal Care and Use Committee and according to the National Institutes of Health guide for the care and use of laboratory animals (protocol approval number 14-539). All animals were housed in rooms with a 12-h light/dark schedule (7 am–7 pm) with food and water available ad libitum. Male and female C57Bl6/J mice were purchased from the Jackson Laboratories (Bar Harbor, ME). The experimental unit for these studies is a single mouse, and all sample sizes, along with statistical analyses used, are reported within the results and/or figures. A surgical control, sham, was used to compare microglial morphology and TMEM119 to healthy tissue while accounting for the general surgical procedure (described below). All mice were randomly assigned to either the sham or stroke condition. The ARRIVE guidelines were used for transparent reporting of research methods and findings [52].

### Postmenopause model

The accelerated ovarian failure model was used to model human postmenopause in this study of ischemic stroke [53, 54]. Similar to previous publications [53, 55], 5-week-old female mice were injected for 21 days with 4-vinylcyclohexene diepoxide (VCD, 160 mg/kg/i.p./day, Millipore Sigma #94956, St. Louis, MO). Follicle depletion and ovarian cessation were assessed ~ 65 days after the first injection via vaginal lavage, and ovarian failure was confirmed by observing 15 days of persistent diestrus prior to tissue collection. Because this is among the first applications of the postmenopause model in ischemic stroke research, we also tested the effect of the VCD injections themselves to exacerbate brain injury after ischemic stroke in male mice. Male mice (C57Bl6/J, 5 weeks) were treated with VCD for 21 days (160 mg/kg/i.p./day) followed by regular animal care until the stroke procedure.

### Ischemic stroke

A transient ischemic stroke was delivered using the filament method as previously published [37, 56]. Ischemic stroke was induced by temporary occlusion of the right middle cerebral artery (MCA) in anesthetized mice (1–2% isoflurane in a 0.4 L/min medical air/0.1 L/min oxygen mixture). All animals were 16 weeks old at the time of ischemic stroke. A filament was advanced, via the internal common carotid artery, to the ostea of the middle cerebral artery. The filament consisted of a blunted 6-0 nylon suture (Ethilon, Ethicon) with a silicone-coated (Xantopren comfort light, Heraeus, New York) tip measuring 0.21–0.25 mm in diameter. The ischemic period for all experiments was 60 min, continuously verified by laser Doppler measures of relative cerebral blood flow to the MCA territory (Perimed Periflux 5000, North Royalton, OH). The sham procedure included all elements up to filament placement. To be included in this study, animals must have experienced a decrease in relative cerebral blood flow of at least 70% of baseline (ischemia) accompanied by reperfusion defined as a return of relative cerebral blood flow to within 30% of baseline. Following 24 h of reperfusion, tissue was immediately collected for infarct analysis or was exsanguinated and perfused with 0.01 M phosphate-buffered saline. For immunohistochemistry (IHC), brain tissue was removed and fixed in 4% paraformaldehyde for 24 h followed by a 30% sucrose solution for 72 h. For western blot methods, brain tissue was removed and rapidly frozen using 2-methylbutane (Thermo Fisher Scientific, Cat# O3551-4) at − 50 °C to prevent tissue cracking. All tissue was stored at − 80 °C until use.

Infarct analysis was carried out using 2,3,5-triphenyltetrazolium chloride (TTC) solution and analysis methods as previously described in detail [57]. Briefly, fresh brain tissue was sliced into sequential 2-mm coronal sections and stained with a 4% TTC solution for 20 min at 37 °C followed by overnight incubation in paraformaldehyde. Tissue sections were then scanned (600 pixels per inch) and saved as .tiff files prior to infarct analysis. The infarcted brain regions were noted as white tissue while healthy regions remained red. To quantify infarct, the percent area of the infarcted regions were quantified using ImageJ tools as well as corresponding contralateral regions; anatomical areas were identified using a mouse stereotaxic atlas as a reference [58]. Total infarct volume is reported as a percentage of the contralateral hemisphere [57].

### Immunohistochemistry

Fixed tissue was sectioned into 50-μm coronal sections (Leica cryostat CM1850, Buffalo Grove, IL) and stored at − 20 °C in a cryoprotectant solution (50% 50 mM PBS, 30% ethylene glycol, 20% glycerol) until IHC experiments. A random and unbiased selection of tissue sections between bregma 0 and + 1 corresponding to ~ 4 mm from the frontal pole was the basis for IHC methods and quantification of microglia morphology, the area of complement receptor 3 (CR3) immunofluorescence, and ionized calcium-binding adaptor molecule 1 (IBA1) and TMEM119 immunofluorescence. Free-floating brain sections were first blocked in 10% horse serum (Vector Laboratories, S-2000-20, Burlingame, CA) and buffer solution (0.01 M PBS, 0.05% Triton, and 0.04% NaN_3_) for 1 h followed by a 72-h incubation with primary antibodies as appropriate: rabbit anti-IBA1 at 1:1000 (Wako, 019-19741, Madison, WI), rat anti-CD11b at 1:500 (Thermo Fisher Scientific RM2800, Waltham, MA), rat anti-CD18 (Millipore MABT42, Danvers, MA), and rat anti-TMEM119 at 1:250 (abcam 209064, Cambridge, UK) [37, 56]. To measure the full CR3 complex, both α and β subunits, antibodies for CD18 and CD11b, respectively, were used in the same incubation. The TMEM119 antibody has been validated for its specificity for microglia and its use in health and disease in an IHC preparation by others [38, 45]. In this case, a healthy brain is considered a positive control, as well as a comparison group. The following are used for a 4-h incubation of 1:250 secondary antibodies (Jackson ImmunoResearch Laboratories, West Grove, PA): donkey anti-rabbit Alexa 488 (711-546-152) and donkey anti-rat Alexa 594 (712-585-150). All tissue was incubated in solutions common to all groups to avoid group/batch differences. All reactions were carried forward at room temperature; washes between incubations were with 0.01 M PBS for 15 min. Vectashield (Vector Laboratories, H-1000) was used to coverslip mounted tissue.

### Imaging

Images were acquired on a confocal microscope (Zeiss NLO 880, San Diego, CA) in the tissue regions in proximity to the infarct area. A × 40 objective (236.16 × 236.16-μm area) was used for the morphology and CR3 percent area analysis while a × 20 objective (473.33 × 473.33-μm area) was used for the percent area IBA1 and TMEM119 analysis. *Z*-stacks were compressed to a 2D image using maximum intensity, channel split, and files saved as .tiff files using the ImageJ software (NIH, v1 53c). Thresholding for the area of CR3-, IBA1-, and TMEM119-positive immunofluorescence was carried out using ImageJ and based on sham images; threshold parameters were consistent for all images. The percent area was recorded for all parameters. The number of cell somas was counted in the IBA1 channel, an approximation of the number of cells imaged in each frame, and all percent area data was divided by this cell count.

### Microglia morphology analysis

The extent of ramified microglial morphology was quantified using an objective and computer-aided skeleton analysis method as previously published in detail [51]. A series of ImageJ plugins (i.e., adjust brightness, unsharp mask, and despeckle) were temporarily applied in order to ensure adequate cell process visualization before the conversion to binary and skeletonized images. The skeletonized representations of original photomicrographs were used for data collection of two parameters: the number of endpoints/cell and process length/cell. The AnalyzeSkeleton (2D/3D) plugin (developed and maintained by Arganda-Carreras et al. [59]) was used to tag elements of microglia skeletons as processes (orange slab voxels) and endpoints (blue) for data collection. We summarized the number of endpoints and process length from AnalyzeSkeleton (2D/3D) plugin data output, and all data were divided by cell soma counts, an approximation of the number of cells imaged in each frame. In addition to the skeleton analysis, additional morphometrics were collected on individual microglia to complement the frame-based analysis using the plugin FracLac for ImageJ [60]. The specifics of this method have been previously described in detail [51]. Three cells were randomly identified and isolated from the saved binary files (described above) for analysis by using a grid and random number generator. Additional structures that abut and surround each cell were manually excluded from the analysis using a Wacom tablet. The ImageJ plugin 3D viewer and original photomicrographs were used to facilitate this process. Binary cells were then converted to outlines using ImageJ before using FracLac for ImageJ for data collection. Using this plugin, we report morphometrics for fractal dimension, a measure of cell complexity; density, a measure of cell size; and span ratio, a measure of cell elongation. For 2D cell outlines, fractal dimension values range from 1 to 2, where 1 summarizes the complexity of a simple circle. These calculations and relationships are best summarized in the reference guide provided for FracLac for Image J (https://imagej.nih.gov/ij/plugins/fraclac/FLHelp/BoxCounting.htm) and additional associated references [60, 61]. Individual cell morphology data was averaged for each parameter to obtain a representative value for each animal. All morphometric analyses were carried out by researchers blinded to the sex group.

### Western blot

In a different cohort of mice, tissue was collected after the ischemic stroke procedure, immediately frozen, and stored until sectioning. Frozen tissue was sectioned into 2-mm coronal sections, and a 1-mm diameter biopsy tool (Harris Micro-punch, Millipore-Sigma, Z708658) was used to collect tissue punches in the ipsilateral regions distal and proximal to the infarcted tissue similar to the IHC regions (Fig. 3b); sham and contralateral tissues were also collected in regions matching the ipsilateral regions. To extract protein, all samples were homogenized in ice-cold lysis buffer (20 mM Tris, 150 mM NaCl, 0.05% Tween) and 1% each of 1 mmol/l PMSF (Sigma-Aldrich, P7626), 200 mM Na3VO4 (Sigma-Aldrich, S6508), and a protease inhibitor cocktail (Sigma-Aldrich, P8340) followed by 2-h digestion at 4 °C. The supernatant was collected after 15 min at centrifugation (14K x G) and analyzed for protein concentration using the Bicinchoninic Acid Kit (Thermo Fisher Scientific, #PI208609, IL,).

To quantify IBA1 and TMEM119 expression in each sample, 15 μg of protein was separated on a 10% Criterion® TGX™ Precast Protein Gel (BioRad Laboratories, 56671034, Hercules, CA) and transferred to a nitrocellulose membrane using a Trans-Blot TurboTransfer System (Bio-Rad Laboratories). A Chameleon® Duo Pre-stained Protein Ladder (LI-COR, 928-60000, Lincoln, NE) was used for size reference, and all blots were scanned on an Odyssey CLx imaging system. Total protein was calculated for each sample following incubation, according to the manufacturer’s specifications, in Revert 700 Total Protein Stain (LI-COR, 926-11010). Non-specific binding was blocked with Odyssey Blocking Buffer (LI-COR, 927-50000) for 1 h at room temperature. The membrane was then cut between the 30- and 25-kDa ladder (Chameleon Duo, 928-60000), and the top membrane with the larger proteins was incubated with rabbit anti-TMEM119 (1:500, Proteintech, 66948-1, IL) while the bottom portion was incubated with rabbit anti-IBA1 antibody (1:1000, Wako, 016-20001). Primary incubation was overnight at 4 °C with a solution of LI-COR Odyssey Blocking Buffer and 0.2% Tween. Each membrane was washed (TBS-T and 0.2 Tween) and then incubated with IRDye® 800 CW goat anti-rabbit secondary antibody (1:10,000, LI-COR, 925-3221) diluted in Odyssey Blocking Buffer and 0.2% Tween for 1 h at room temperature. Total protein and bands at ~ 45 kDa and ~ 17 kDa for TMEM119 and IBA1, respectively, were analyzed with the Empiria Studio Software according to the manufacturer’s recommendations. All densitometry measurements were normalized to total protein prior to statistical analysis. All analysis was carried out by researchers blinded to the sex group.

Examples of positive and negative control for TMEM119 were run with the same conditions, but on a 10% Mini-Protean® TGX™ Precast Protein Gel (Supplemental Figure 1, BioRad, 4561036). Because of its limited use, western analysis was used to test TMEM119 for its specificity using 3 μg of protein extracted from SH-SY5Y cells, mouse brain cortex homogenate, and mouse spleen homogenate. The SH-SY5Y cells (ATCC, VA) are a commonly used human-derived neuroblastoma cell line and were the particular cell line depicted by the manufacturer (https://www.ptglab.com/products/TMEM119-Antibody-27585-1-AP.htm). In this case, TMEM119 should be present in the SH-SY5Y and brain sample but not in the spleen. On the other hand, IBA1 should be present in all samples. In Supplemental Figure 1 we show the expected TMEM119 band at ~ 47 kDa in the SH-SY5Y lane. Also, we show that while TMEM119 is present in the SH-SY5Y and brain samples, it is absent in the spleen sample. On the other hand, IBA1 is present in all three samples, SH-SY5Y, spleen, and brain samples.

### Statistical analysis

Data are shown as mean ± SEM and were analyzed using the GraphPad Prism 6.0 software (GraphPad Software, La Jolla, CA). Differences in infarct size, morphology, phagocytosis, TMEM119 immunohistochemistry, and western blot were analyzed using non-repeated measure two-way analysis of variance (ANOVA) followed by a two-tailed Sidak multiple comparisons tests, or one-way ANOVA with Bonferroni post hoc test as appropriate.

## Results

**Fig. 1:**
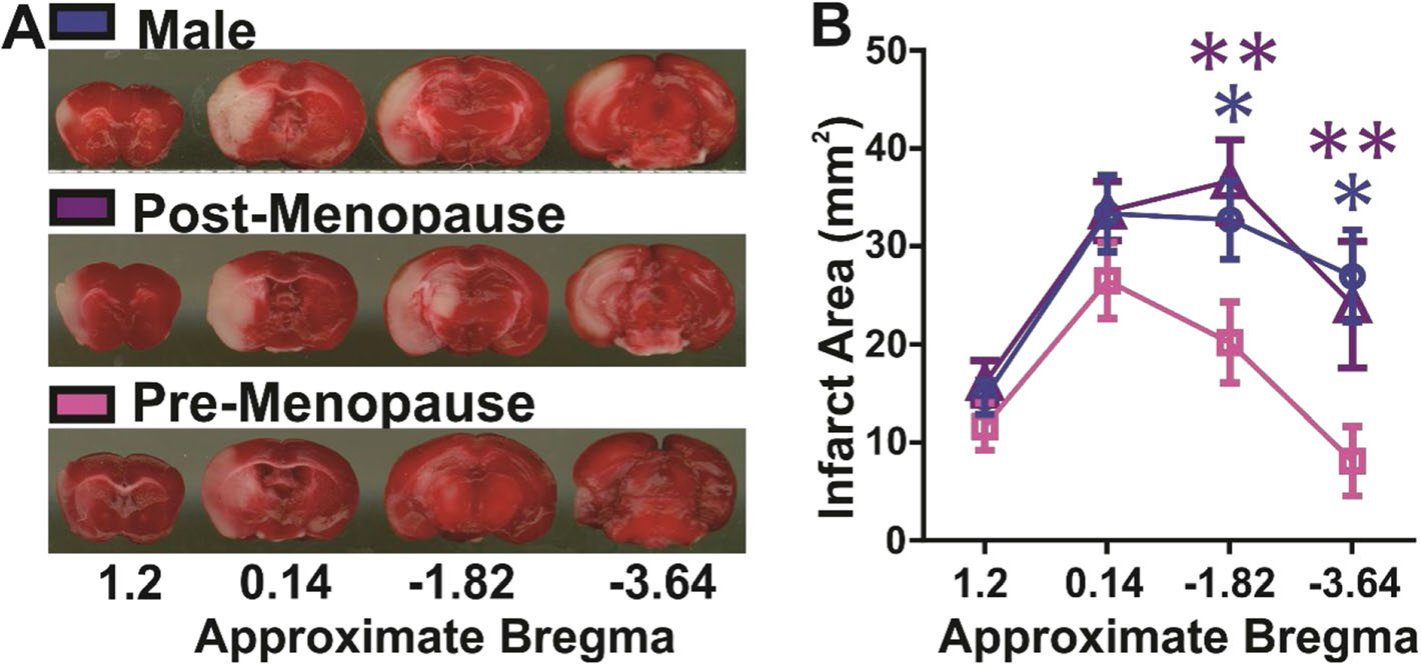
Brain infarct size is increased in male and postmenopausal mice vs. premenopausal mice after ischemic stroke. **a** Images of 2,3,5-triphenyltetrazolium chloride (TTC)-stained brain sections in male and female postmenopause and premenopause mice after 60 min of ischemic stroke and 24 h of reperfusion. The white area is necrotic, and the red area is healthy tissue. **b** Summary data of infarct area (mm^2^) in a region between approximately 1.2 and − 3.64 bregma. The brain infarct area is increased in male or postmenopause mice vs. premenopause mice. *p < 0.05, **p < 0.01. Male, *n* = 13; postmenopause female, *n* = 11; premenopause female, *n* = 10

Representative images (Fig. 1a) and summary data (Fig. 1b) illustrate the distribution of brain infarct in the right hemisphere 60 min post-ischemia and 24 h after reperfusion between the sex groups. Here, we show that infarct size is increased in male and postmenopause female mice vs. premenopause mice (repeated measure two-way ANOVA: section: *F*_(3, 93)_ = 22.5, *p* < 0.0001; sex: *F*_(2, 31)_ = 4.9, *p* < 0.01; interaction *F*_(6, 93)_ = 1.9, *p* < 0.05). Differences in the infarct size between the sex groups occur in the rostral brain regions (6 mm and 8 mm), but not caudal. All post hoc analyses are reported in Fig. 1b. We show that VCD, delivered i.p. when mice are 5–8 weeks of age, does not have an effect on brain infarct size after an ischemic stroke delivered at 16 weeks of animal age (Supplemental Figure 2, repeated measure two-way ANOVA: section: *F*_(3, 15)_ = 13.04, *p* < 0.001; VCD treatment: *F*_(1, 5)_ = 2.59, *p* > 0.05; interaction: *F*_(3, 15)_ = 0.12, *p* > 0.05).

**Fig. 2:**
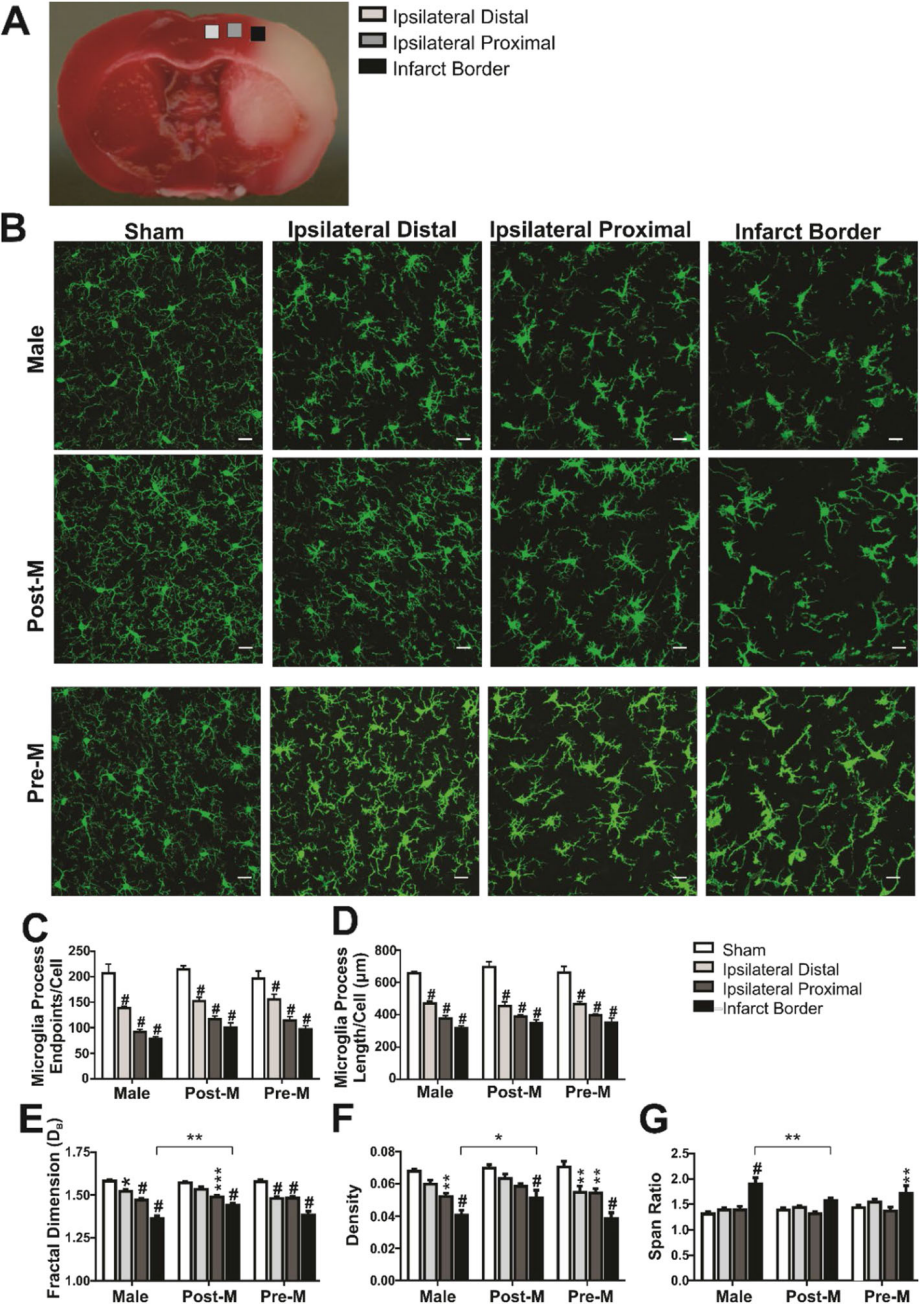
Microglia morphologic response to ischemic stroke is not influenced by sex or menopause. **a** Images of IBA1-positive microglia (× 40 objective) in the cortex of mice after sham and stroke procedure. Three cortical ipsilateral regions in proximity to the infarcted tissue were included in the analysis as depicted in **b**. Summary data of skeleton analysis show that both the number of microglia process endpoints/cell (**c**) and summed process length/cell (**d**) decrease in proximity to the brain injury but with no sex differences. Fractal analysis of individual cells shows that fractal dimension (**e**) and density (**f**) are, like skeleton analysis, decreasing in proximity to the brain injury and with sex differences noted between the male and postmenopause group in the proximal region. On the other hand, span ratio (**g**) was consistent among most regions and sex groups but was dramatically increased in the proximal region of all sex groups. Post hoc analysis is shown in the figure (*p < 0.05, **p < 0.01, ***p < 0.001, and ^#^*p* < 0.0001 vs. sham region; sex differences are noted by the bracket: *p < 0.05 and **p < 0.01). Male, *n* = 6; postmenopause female, *n* = 6; premenopause female, *n* = 6 for all regions. Scale bar = 20 μm

We next examined the contribution of sex and menopause on the morphologic and phagocytic responses of microglia to ischemic stroke. Total infarct volume was reduced in premenopause females as compared to male (*p* < 0.05) and postmenopausal female (*p* < 0.05) mice. However, at 4 mm from the frontal pole, infarct size was not significantly different across the sex groups (one-way ANOVA, *p* = 0.3). Therefore, to avoid the confounding variable of infarct size differences among the groups, all histological measures of microglial responses were assessed in coronal tissue sections 4 mm from the frontal pole, corresponding to bregma 0 to + 1. Figure 2a shows the brain regions imaged using a × 40 objective for microglia morphology analysis. Figure 2b illustrates the IBA1-positive microglia imaged in each brain region. We measured the changes in microglia morphology among the sex groups by quantifying the number of microglia endpoints and process length per cell via the IHC and ImageJ analysis techniques. We show that the process endpoints on microglia decrease in the regions that are proximal to the infarcted tissue similarly among the sex groups (region: *F*_(3,60)_ = 78.99, *p* < 0.0001; sex: *F*_(2, 60)_ = 3.0, *p* = 0.06; interaction: *F*_(6, 60)_ = 1.0, *p* > 0.05). This effect is also observed for the summed process length per cell (region: *F*_(3, 60)_ = 115.7, *p* < 0.0001; sex: *F*_(2, 60)_ = 0.46, *p* > 0.05; interaction: *F*_(6, 60)_ = 0.33, *p* > 0.05). We also show that individual cell analyses were similar to the higher-throughput frame-based skeleton analysis, however with more sensitivity to detect differences among the sex groups. Microglia fractal dimension decreased in proximity to the infarcted tissue and according to the sex group with the only sex difference noted in the proximal region between the male and postmenopause female groups (region: *F*_(3, 60)_ = 86.04, *p* < 0.0001; sex: *F*_(2, 60)_ = 3.8, *p* < 0.05; interaction: *F*_(6, 60)_ = 2.5, *p* < 0.05). Microglia density showed a similar decrease across the regions and sex groups (region: *F*_(3, 60)_ = 35.0, *p* < 0.0001; sex: *F*_(2, 60)_ = 3.7, *p* < 0.05; interaction: *F*_(6, 60)_ = 0.27, *p* > 0.05). In contrast to all other morphometric parameters, microglia span ratio was only increased in the proximal brain region and did not vary across the sex groups (region: *F*_(3, 60)_ = 18.12, *p* < 0.0001; sex: *F*_(2, 60)_ = 0.17, *p* > 0.05; interaction: *F*_(6, 60)_ = 0.08, *p* > 0.05). All post hoc analyses are reported in Fig. 2c–g (*p < 0.05, **p < 0.01, ***p < 0.001, and ^#^*p* < 0.0001 vs. sham region; sex differences are noted by the bracket: *p < 0.05 and **p < 0.01).

**Fig. 3:**
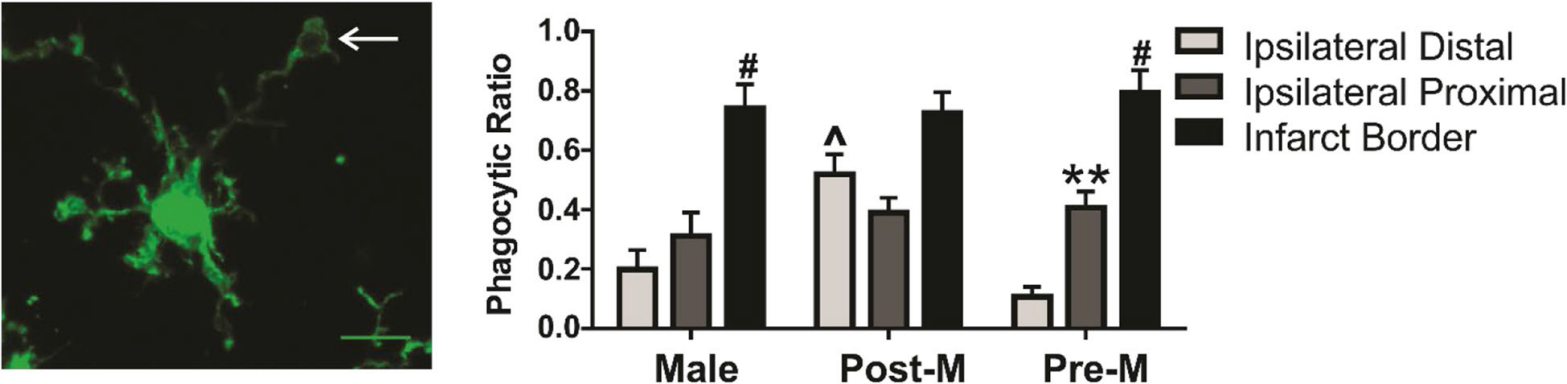
Microglia phagocytic responses are influenced by proximity to ischemic stroke, sex, and menopause. An example of microglia phagosome morphology (white arrow) and summary data of microglia phagocytic ratio (phagocytic cells/total microglia cells in the image frame) in ipsilateral hemisphere regions after ischemic stroke. Phagocytic ratio is increased in proximity to the infarcted region and is different according to sex and menopause. Regional post hoc analyses (vs. ipsilateral distal region **p < 0.01, ****p < 0.0001) and sex group (^*p* < 0.01 vs. male mice and *p* < 0.001 vs. premenopause female mice) are reported in the figure. Male, *n* = 6; postmenopause female, *n* = 6; premenopause female, *n* = 6

To assess phagocytosis, we quantified the number of microglia with phagosomes as well as the presence of phagocytic receptor CR3. Phagosomes were identified by the ball and chain morphology shown in Fig. 3 from the series of IBA1-positive images. Our data show that microglia phagosomes are more present in the brain region that borders the necrotic tissue in all sex groups. However, sex and menopause influence microglia phagosomes differently in the distal and proximal brain regions (region: *F*_(2, 45)_ = 44.54, *p* < 0.0001; sex: *F*_(2, 45)_ = 3.31, *p* < 0.05; interaction: *F*_(4, 45)_ = 4.22, *p* < 0.01). All post hoc analyses are reported in Fig. 3 (**p < 0.01 and ^#^*p* < 0.0001 vs. sham region; ^*p* < 0.05 vs. male).

**Fig. 4:**
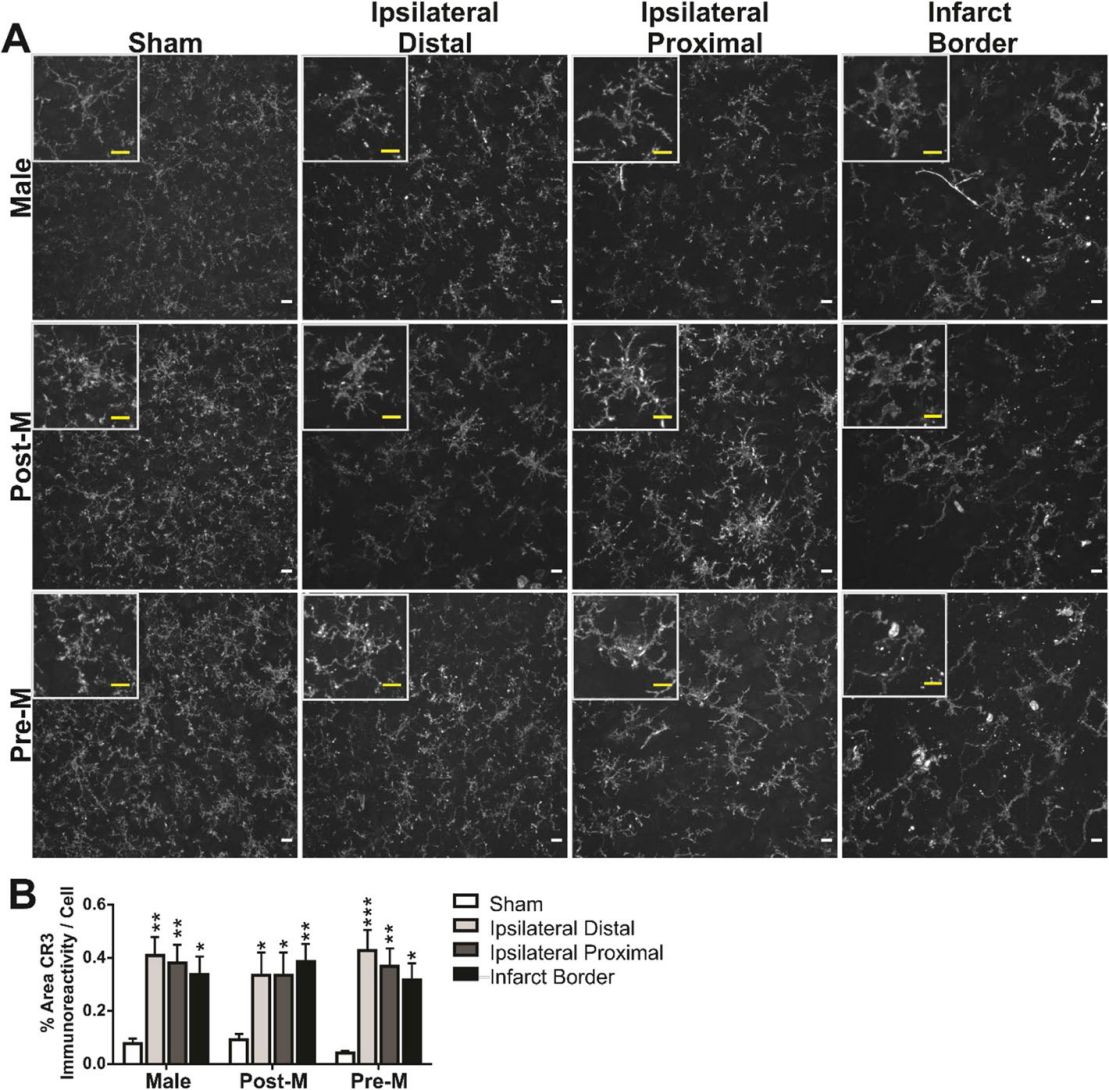
The percent area of CR3 per cell is increased in the ipsilateral hemisphere following ischemic stroke similarity among the sex groups. **a** Images of CR3 immunofluorescence (× 40 objective) in the cortex of mice after sham and stroke procedure. **b** Summary data show that the percent area of CR3 per cell is increased, similarly, among the ipsilateral regions when compared to sham, an observation that was similar among the sex groups. Regional post hoc analyses (vs. sham region *p < 0.05; **p < 0.01, ***p < 0.001) are reported in the figure. Male, *n* = 6; postmenopause female, *n* = 6; premenopause female, *n* = 6

The percent area of CR3 immunofluorescence was collected in the same regions illustrated in Fig. 2a, in a different fluorescence channel. Figure 4a illustrates the area of CR3 immunofluorescence on microglia imaged in each brain region and sex group; cropped inset images show the detail. We measured the changes in CR3 immunofluorescence per cell in 8-bit images by using ImageJ analysis techniques and consistent thresholding parameters (20–255). Figure 4b summarizes the findings that the percent area of CR3 immunoreactivity is increased from sham in the ipsilateral hemisphere after ischemic stroke similarly among the sex groups (region: *F*_(3,60)_ = 16.35, *p* < 0.0001; sex: *F*_(2, 45)_ = 0.05, *p* > 0.05; interaction: *F*_(6,60)_ = .89, *p* > 0.05). All post hoc analyses are reported in Fig. 4b (*p < 0.05; **p < 0.01, and ***p < 0.001 vs. sham region).

**Fig. 5:**
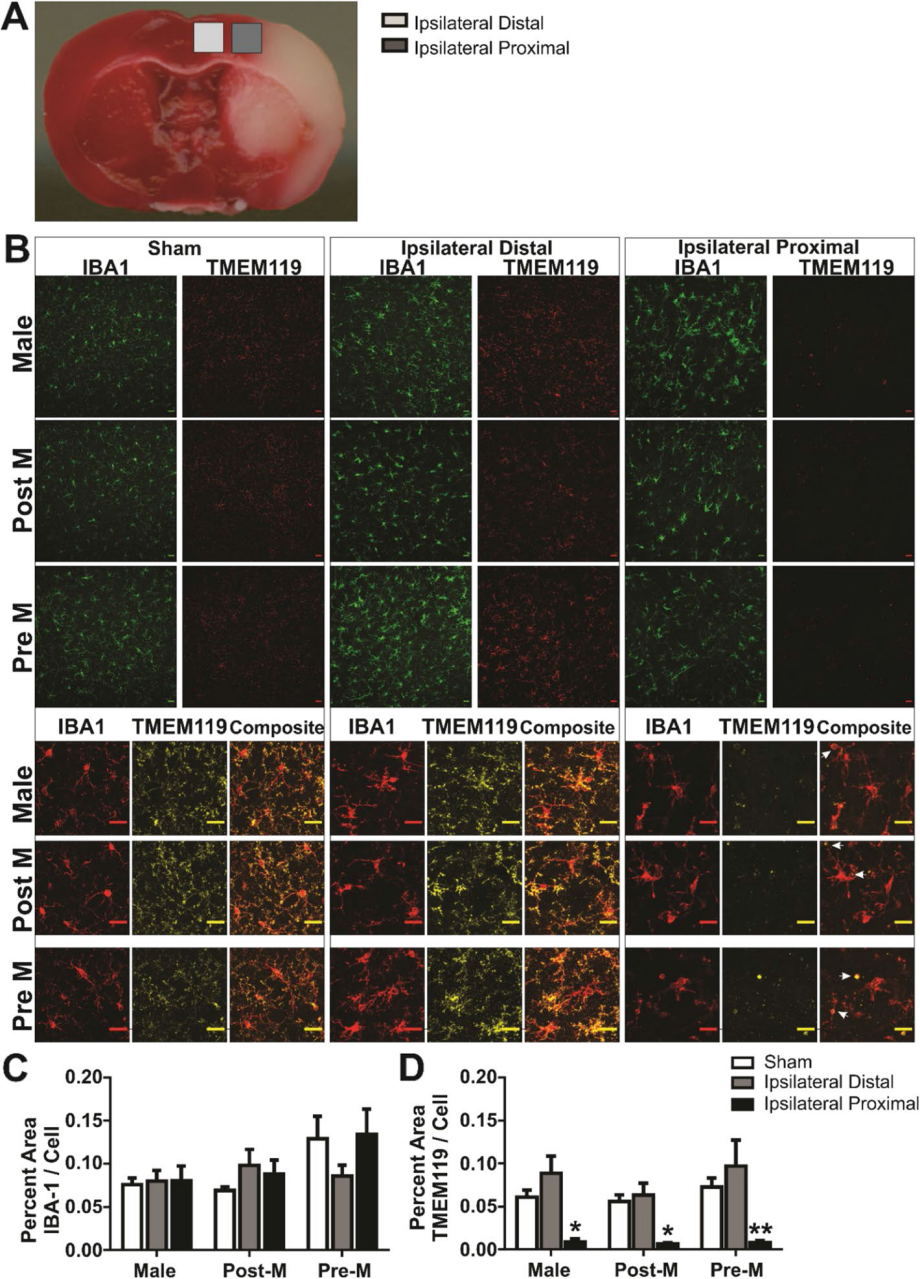
Percent area of TMEM119 immunofluorescence is decreased in proximity to the infarcted tissue. **a** Images of IBA1 and TMEM119 immunofluorescence (× 20 objective) in the cortex of mice after sham and stroke procedure. Two brain regions (distal and proximal to injury) were imaged in the ipsilateral hemisphere. Below, cropped images show the detail, and composite show co-localization of IBA1 and TMEM119 immunofluorescence. **b** Illustration of brain regions imaged. **c** The percent area of IBA1 immunofluorescence per cells in the frame remains unchanged among brain regions and sex. **d** The percent area of TMEM119 immunofluorescence per IBA1 cells in the frame is decreased in the ipsilateral proximal region vs. sham in all sex groups. *p < 0.05; **p < 0.01. *n* = 6–9 in the sex groups and regions. Scale bar = 20 μm

We measured microglia TMEM119 immunofluorescence to assess if a portion of the IBA1-positive cells present in the ipsilateral brain regions after ischemic stroke were infiltrating macrophages. Our interest was specific to the infarct border region because of the decreased ramification and high level of phagosomes that may be indicative of a population of infiltrating macrophages rather than resident microglia. Figure 5a shows the brain regions imaged using a × 20 objective to illustrate TMEM119 and IBA1 immunofluorescence with representative images shown in Fig. 5b; cropped and merged images show the detail and co-localization of the two channels. Although the percent area of IBA1 immunofluorescence per frame is similar among the brain regions and sex groups (region: *F*_(2, 61)_ = 0.43, *p* > 0.05; sex: *F*_(2, 61)_ = 3.57, *p* < 0.05 with no post hoc differences; interaction: *F*_(4, 61)_ = 1.23, *p* > 0.05), TMEM119 immunofluorescence is significantly decreased in the ipsilateral hemisphere proximal brain region with a similar effect observed among the sex groups (region: *F*_(2, 62)_ = 23.51, *p* < 0.0001; sex: *F*_(2, 62)_ = 1.07, *p* > 0.05; interaction: *F*_(4, 62)_ = 0.40; *p* > 0.05). All post hoc results are reported in Fig. 4c, d (*p < 0.05, **p < 0.01 vs. sham region). Arrows in the cropped images in the ipsilateral proximal region highlight the observation that, although decreased, much of the remaining TMEM119 immunofluorescence was colocalized to phagosome-type structures (i.e., microglia processes with a ball and chain morphology).

**Fig. 6:**
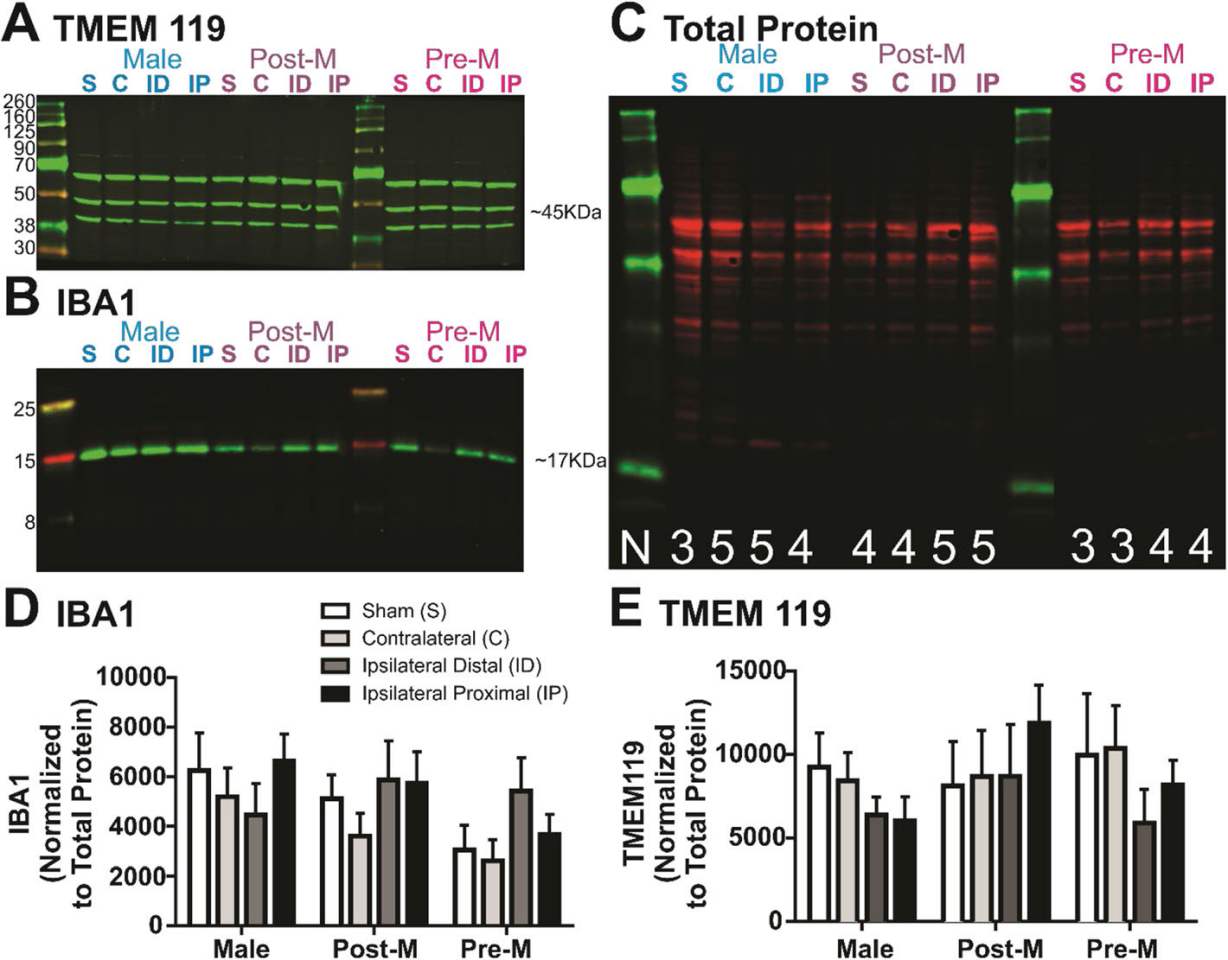
TMEM119 protein expression is unchanged in proximity to the infarcted tissue. Example blots of TMEM119 (**a**), IBA1 (**b**), and total protein (**c**) for the regions and sex groups. **d** Summary of IBA1 (**d**) and TMEM119 (**e**) data illustrate that these proteins remain relatively unchanged among the brain regions and sex groups after ischemic stroke. Sample sizes range from 3 to 5 among the sex groups and regions and are shown in **a**

We further validated our immunofluorescence findings by measuring TMEM119 protein expression using western analysis. Brain tissue punches were carefully extracted from the tissue regions similar to the immunofluorescence regions as shown in Fig. 5a. Figure 6a–c are example blots of total protein, TMEM119, and IBA1, respectively. IBA1 protein expression was similar among the brain regions and sex groups (Fig. 6d; region: *F*_(3, 37)_ = 0.97, *p* > 0.05; sex: *F*_(2, 37)_ = 2.38, *p* > 0.05; interaction: *F*_(6, 37)_ = 0.73, *p* > 0.05). Different than IHC findings, summary data illustrate that TMEM119 protein levels are also similar among the brain regions and sex groups (region: *F*_(3, 37)_ = 0.61, *p* > 0.05; sex: *F*_(2, 37)_ = 0.66, *p* > 0.05; interaction: *F*_(6, 37)_ = 0.61, *p* > 0.05). All post hoc results are reported in Fig. 6d, e.

## Discussion

Our primary purpose was to utilize both morphological analysis and a combination of TMEM119 immunofluorescence and protein expression to distinguish between microglial and infiltrating macrophage populations after ischemic stroke and 24 h of reperfusion. Our secondary purpose was to test for sex differences and, in females, the effect of menopause on microglia/macrophage cell populations after ischemic stroke. We are among the first to apply a postmenopause model that retains intact but follicle-depleted [55] ovaries to study sex differences in ischemic stroke injury and microglial responses. Using this model, we show that stroke infarct size is increased in male and postmenopausal female mice compared to premenopausal mice; this difference primarily occurs in the caudal brain regions. The primary findings of this study are that while microglia become de-ramified in proximity to ischemic stroke, a large portion, if not all, of the imaged cells retained distinct cell processes. In addition, the ramified cells abutting necrotic tissue in the infarct border appear highly phagocytic as evidenced by prominent ball and chain morphologies. The lack of TMEM119 immunofluorescent-positive cells covered an unexpectedly large region that extended beyond the infarct border where infiltrating macrophages were most likely to have been present. Western blot analysis was used to validate the IHC findings. However, we show for the first time that the pattern of TMEM119 expression was not consistent between these methods. In light of these morphological and methodological incongruities, we are unable to definitively distinguish microglia from infiltrating macrophage cell populations in proximity to brain injury 24 h after ischemic stroke using TMEM119 alone. Instead, we suggest that in the context of acute ischemic stroke injury, TMEM119 is not a stable marker to denote microglia from infiltrating macrophages. The secondary findings of this study are that, when controlling for brain injury area, the transition of ramified microglia to less ramified cells was similar among male and pre- and postmenopause sex groups. The percent area of CR3 immunofluorescence, a receptor known for initiating phagocytosis, was significantly increased in response to ischemic stroke similarly among the sex groups. However, we show evidence that phagocytic cells—cells with high phagocytic ratios—are distributed across a larger area in postmenopause mice than the male or premenopause groups in this preclinical model. Lastly, TMEM119 expression, whether measured via IHC or western blot methods, was similar among the sex groups. These findings confirm and expand upon the current body of knowledge regarding post-stroke microglia/macrophage populations, incorporating sex as a biological variable and the consideration of, in females, postmenopause using a model that mimics ovarian failure.

By combining a morphological assessment and a unique microglia marker, such as TMEM119, distinguishing between microglia and infiltrating macrophage populations might be possible in the healthy brain [38]. However, this approach was applied here to an acute brain injury model with gross brain injury, with inconclusive results. Microglia are known to be consistently distributed among the parenchyma with regions demarcated by their non-overlapping ramified morphology [62]. This pattern was observed in the present data with one exception—the region that directly abuts the infarct. In the infarct border region, microglia distribution in the parenchyma becomes somewhat irregular as de-ramified cells were noted to be elongated and in possession of a great many phagosomes when compared to cells in other regions, indicative of a greater degree of phagocytosis proximal to the brain injury. As an additional marker of phagocytosis, CR3 was relatively stable across the brain regions and sex groups in the ischemic hemisphere. However, we recognize as a limitation of this study that other indicators of phagocytosis are available for measurement [63–65] and possible of yielding additional information or increased sensitivity. Future studies will focus on detailing phagocytosis in this proximal brain region where phagocytic activity is abundant.

Current literature suggests that the diapedesis of systemic cells into the parenchyma remains largely localized to the infarct core with little presence in the surrounding tissue at 24 h post-stroke [40, 66]. Therefore, we hypothesized that cells absent of TMEM119 immunofluorescence would be more or less confined to the infarct border region. We found that cells with reduced TMEM119 immunofluorescence area extended well into the proximal and almost to the distal region. In these regions, cells possessed an abundance of processes and were uniformly positioned in the parenchyma—both characteristics of microglia [38]. This led us to verify our IHC findings using western blot methods, which revealed a discrepancy. A few scenarios may account for the conflicting results between methods. First, it is often the case that antibodies successfully used in immunohistochemistry methods are not always successful in western blot methods due to sample preparations. This was the case and a limitation of this study; two different antibodies were assessed, and neither worked in the complementary method. However, we included appropriate positive and negative controls to ensure the validity of these manufacturer-prepared antibodies. Second, it is possible that TMEM119 possesses a functional response to a gross injury such as ischemic stroke. In fact, TMEM119 was not entirely absent from ramified cells in the proximal region and, when present, appeared to colocalize to phagosomes. These data are in parallel to recent findings presented by Li et al. who illustrate that *Tmem119* transcripts are decreased 1 day post-injury in a model of intracerebral hemorrhage [49]. Combined, these observations may suggest a yet undetermined response to injury associated with phagocytosis. Others have demonstrated that, in cultured human microglia, *TMEM119* transcripts are reduced by the presence of the interleukin IL-4 [67] or interferon gamma (INFγ), but not LPS [48], which could indicate an injury-specific transcriptional change.

Transmembrane proteins are part of a large family that, in general, consist of anchored proteins thought to act as transport channels across the lipid bilayer [46, 47]. However, by and large, the exact functions of these abundant proteins, including TMEM119 [45], remain unknown [47]. TMEM119 was originally identified as an osteoblast inhibitory factor (OBIF) derived from osteoclast cell lines [44, 68]. Notably, osteoclasts are cells derived from the monocyte/macrophage cell line [69]. *Obif* mRNA is present in a diversity of tissues (https://www.ncbi.nlm.nih.gov/gene/231633), and while Mizuhashi et al. [41] describes bone mineralization and spermatogenesis phenotypes in *Obif*^−/−^ mice, a central nervous system phenotype was not investigated. Others have demonstrated that TMEM119 has an important function in the differentiation of myoblasts into osteoblasts [43, 48, 70–73]. Investigations of TMEM119 in the brain center around novel methodological tools to distinguish microglia from other myeloid populations [38, 74] and descriptions of cell populations in health and disease [45, 48, 50, 75, 76]. Despite these recent increases in utilizing TMEM119 in brain research, its function in the brain remains unknown. In our images, TMEM119 was rarely completely absent from IBA1-positive cells, but rather, the area of immunofluorescence was visibly diminished and, where present, often co-localized to microglia phagosomes or processes. This observation suggests to us that either the TMEM119 epitope used for antibody-antigen interactions in IHC preparations may be degraded with injury or that this transmembrane protein has a functional response to injury that must be further characterized. Similar to another microglia distinguishing protein purinergic receptor P2Y12 [77], TMEM119 expression may change according to the physiologic or pathologic stimuli or to the local inflammatory environment. Therefore, its methodological application may be most useful in discriminating among populations of microglial responses to injury rather than distinguishing among myeloid populations. A limitation of this study is the single time point of data collection. Microglia and macrophages are dynamic cells that undergo a dramatic transformation in terms of gene expression and phenotypes after ischemic brain injury [40]. In fact, others have shown that reprogrammed macrophages may become ramified over time, first identified at a 30-day time point in a model of neonatal stroke. However, the majority of identified infiltrating macrophages were round-shaped at earlier time points [40]. It is plausible that the detectability of TMEM119 may also vary at different time points after stroke. To this point, *Tmem119* transcripts were recently shown to vary over time in a model of intracerebral hemorrhage [49]. Therefore, a time course of TMEM119 protein expression in adult stroke would be a valuable investigation as a qualitative marker of microglia during both the acute and chronic stages of ischemic stroke.

It is well established that sex is an important biological variable in determining ischemic stroke outcomes [7, 29]. However, the mechanisms that underlie observed sex differences in brain injury outcomes are not fully established. Therefore, we included the three relevant sex groups, pre- and postmenopause females as well as males. We posited that differences in cell populations (microglia vs. infiltrating macrophages) among these sex groups would be insightful to understand disparate injury evolution and stroke outcomes. We used a postmenopause model that induced ovarian failure rather than ovariectomy to best represent the human population, where surgically induced menopause is less common than naturally occurring menopause. Using this model, we replicated the large body of previous research, primarily carried out in an ovariectomized rodent models, that male and postmenopause female mice have more severe stroke injury when compared to premenopausal female mice [7]. However, infarct size was similar between age-matched postmenopause female mice and male mice. By using a limited region of interest for our investigation, we were able to control for infarct size in the immediate region of our investigation. Our findings suggest that sex and menopause are not factors that influence the morphological response of microglia to ischemic stroke injury or post-stroke microglia TMEM119 protein expression. Considering the similarity in the findings across groups, whether it be microglia or macrophages, cell responders appear to be similar regardless of sex or menopause status in this brain region where infarct size is relatively constant. However, a limitation of this study is that age, a confounding variable to postmenopausal status in women, was not included in this study. Others have illustrated the important aspect of age on microglia homeostasis and responses to injury [78–82] that may be experienced differently in males and females.

## Conclusions

We demonstrate that the accelerated ovarian failure model, wherein mouse ovaries remain intact but dysfunctional, can be utilized to investigate ischemic stroke pathophysiology. Using this model, we illustrate that microglia transition into less ramified cells when in proximity to the infarct but without a sex or menopause effect. On the other hand, the phagocytic nature of microglia indicated by morphology is increased in regions that are distal to the infarcted region in postmenopause mice when compared to the other sex groups and may contribute to disparities in post-stroke injury. This is an area for future investigation. While TMEM119 remains a promising addition to the repertoire of markers used to study microglia, the results of this study demonstrate that it cannot be used to distinguish between resident microglia and infiltrating macrophages in all cases. The disparity between IHC and western analysis suggests that the function of TMEM119 is more nuanced than initially thought and that its efficacy as a reliable microglial marker may be both function- and response-specific. Furthermore, the apparent localization of TMEM119 immunofluorescence to microglial processes, and particularly to phagosomes when present in proximity to injury, suggests that its function may be specific to filopodia extension, cell mobility, and an actively phagocytic microglial response.

## Supplementary Information

**Additional file 1: Supplemental Figure 1.** TMEM119 antibody validation in control samples. Example blots of TMEM119 (A), IBA1 (B), and total protein (C) in SH-SY5Y cells (lane 1), spleen (lane 2) and brain (lane 3) samples. This image illustrates that while TMEM119 is present in the SH-SY5Y cells (tested by manufacturer) and brain samples, it is not present in the spleen. IBA1 is present in all samples.

**Additional file 2: Supplemental Figure 2.** VCD injections do not affect stroke size in male mice. A) Images of 2,3,5-triphenyltetrazolium chloride stained brain sections in male mice treated with saline and male mice treated with VCD after 60 min of ischemic stroke and 24 h of reperfusion. White area is necrotic and red area is healthy tissue. B) Summary data of infarct area (mm^2^) between approximately 1.2 and -3.64 bregma. Brain infarct area is not significantly different between saline and VCD treated mice. Sample size: Male saline n = 4, Male VCD n = 3.

**Additional file 3.**

## Data Availability

The datasets used during the current study are available from the corresponding author on reasonable request.

## Abbreviations

TMEM119: Transmembrane protein 119
VCD: 4-Vinylcyclohexene diepoxide
MCA: Middle cerebral artery
IBA1: Ionized calcium-binding adaptor molecule 1
ANOVA: Analysis of variance
IHC: Immunohistochemistry
IL-4: Interleukin 4
INFγ: Interferon gamma
LPS: Lipopolysaccharide
OBIF: Osteoblast inhibitory factor
