# Supplementary figures and images for: Can quantifying morphology and TMEM119 expression distinguish between microglia and infiltrating macrophages after ischemic stroke and reperfusion in male and female mice?
Source: J Neuroinflammation. 2021 Feb 22;18:58. doi: 10.1186/s12974-021-02105-2 (PMC7901206; doi:10.1186/s12974-021-02105-2)

Supplemental Figure 1.

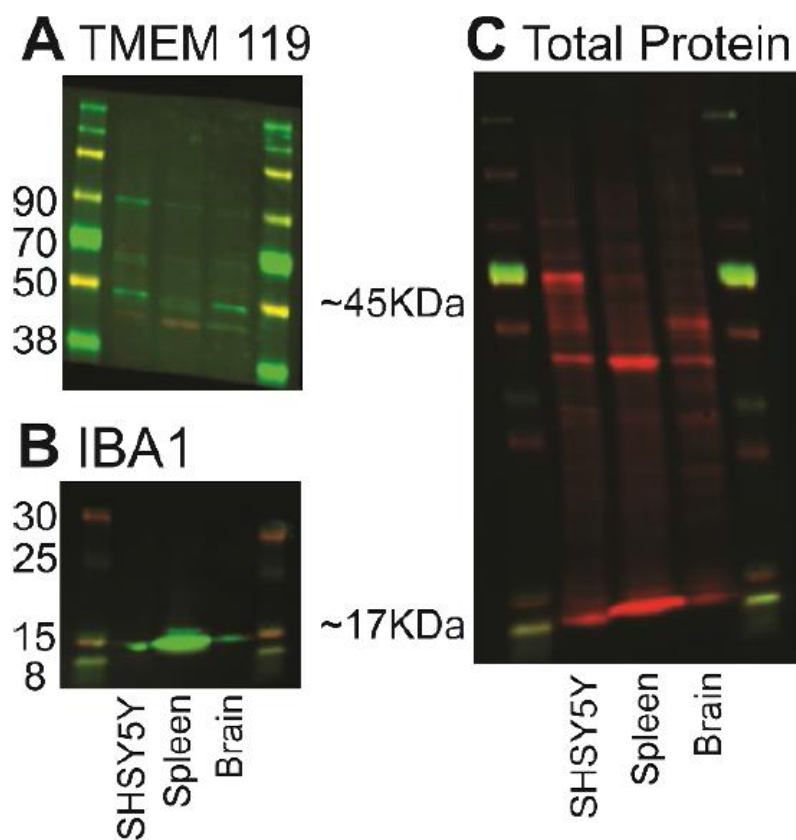

Supplement: Supplementary file 1 — Additional file 1: Supplemental Figure 1. TMEM119 antibody validation in control samples. Example blots of TMEM119 (A), IBA1 (B), and total protein (C) in SH-SY5Y cells (lane 1), spleen (lane 2) and brain (lane 3) samples. This image illustrates that while TMEM119 is present in the SH-SY5Y cells (tested by manufacturer) and brain samples, it is not present in the spleen. IBA1 is present in all samples. [file 12974_2021_2105_MOESM1_ESM.pdf]

Supplemental Figure 2.

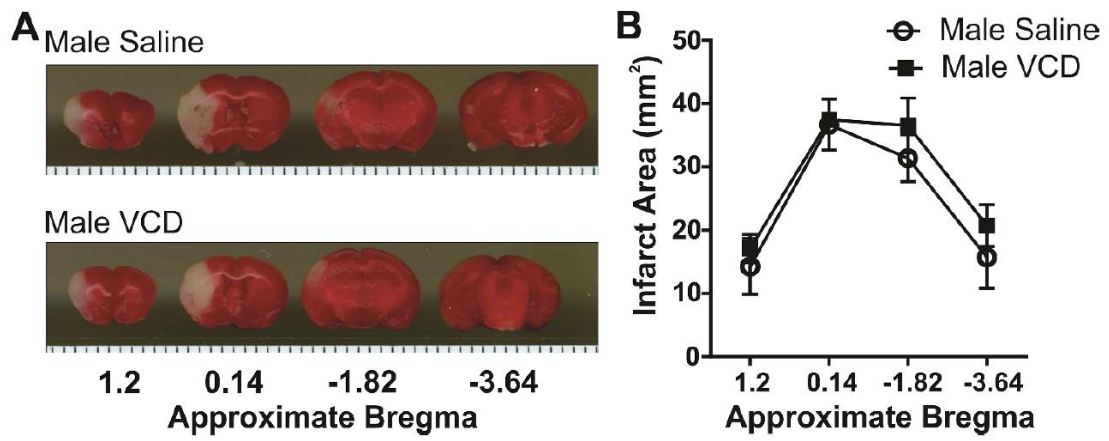

Supplement: Supplementary file 2 — Additional file 2: Supplemental Figure 2. VCD injections do not affect stroke size in male mice. A) Images of 2,3,5-triphenyltetrazolium chloride stained brain sections in male mice treated with saline and male mice treated with VCD after 60 min of ischemic stroke and 24 h of reperfusion. White area is necrotic and red area is healthy tissue. B) Summary data of infarct area (mm2) between approximately 1.2 and -3.64 bregma. Brain infarct area is not significantly different between saline and VCD treated mice. Sample size: Male saline n = 4, Male VCD n = 3. [file 12974_2021_2105_MOESM2_ESM.pdf]
