# Supplementary material for: Can quantifying morphology and TMEM119 expression distinguish between microglia and infiltrating macrophages after ischemic stroke and reperfusion in male and female mice?
Source: J Neuroinflammation. 2021 Feb 22;18:58. doi: 10.1186/s12974-021-02105-2 (PMC7901206; doi:10.1186/s12974-021-02105-2)
Supplement: Supplementary file 3 — Additional file 3. [file 12974_2021_2105_MOESM3_ESM.pdf]

Supplemental Table 1.

	Male (16 week)	Postmenopause (16 week)	Premenopause (16 week)	Total
**Figure 1**	13	11	10	34
**Figure 2**				
sham	6	6	6	
ipsilateral distal	6	6	6	
ipsilateral proximal	6	6	6	
Infarct border	6	6	6	
**Figure 3**				
ipsilateral distal	6	6	6	
ipsilateral proximal	6	6	6	
Infarct border	6	6	6	
**Figure 4**				
sham	6	6	6	
ipsilateral distal	6	6	6	
ipsilateral proximal	6	6	6	
**Figure 5**				
sham	6	6	6	
ipsilateral distal	6	6	6	
ipsilateral proximal	6	6	6	18
ALL IHC DATA WERE COLLECTED FROM THE SAME ANIMALS				
**Figure 6**				
sham	**3**	**4**	**3**	
contralateral	**5**	4	3	
ipsilateral distal	5	**5**	**4**	
ipsilateral proximal	4	5	4	24
BOLDED VALUE REPRESENTS ANIMALS; TISSUE LYSATE WAS NOT AVAILABLE FOR ALL SAMPLES, DECREASING SAMPLE SIZE IN SOME CASES				
**Supplemental Figure 2**	Male (16 week)			
Saline treated	4			
VCD treated	3			
Total	7			
TOTAL ANIMAL USE				83
